# Development of a multivariable prediction model for plantar foot ulcer recurrence in high-risk people with diabetes

**DOI:** 10.1136/bmjdrc-2020-001207

**Published:** 2020-03-18

**Authors:** Wouter B aan de Stegge, Ameen Abu-Hanna, Sicco A Bus

**Affiliations:** 1 Department of Rehabilitation Medicine, University of Amsterdam, Amsterdam UMC, Amsterdam, The Netherlands; 2 Department of Medical Informatics, Universitiy of Amsterdam, Amsterdam UMC⁠, Amsterdam, The Netherlands

**Keywords:** foot plantar ulcers, prediction, biomechanics

## Abstract

**Introduction:**

Forty per cent of people with diabetes who heal from a foot ulcer recur within 1 year. The aim was to develop a prediction model for plantar foot ulcer recurrence and to validate its predictive performance.

**Research design and methods:**

Data were retrieved from a prospective analysis of 171 high-risk patients with 18 months follow-up. Demographic, disease-related, biomechanical and behavioral factors were included as potential predictors. Two logistic regression models were created. Model 1 for all recurrent plantar foot ulcers (71 cases) and model 2 for those ulcers indicated to be the result of unrecognized repetitive stress (41 cases). Ten-fold cross-validation, each including five multiple imputation sets, was used to internally validate the prediction strategy; model performance was assessed in terms of discrimination and calibration.

**Results:**

The presence of a minor lesion, living alone, increased barefoot peak plantar pressure, longer duration of having a previous foot ulcer and less variation in daily stride count were predictors of the first model. The area under the receiver operating curve was 0.68 (IQR 0.61–0.80) and the Brier score was 0.24 (IQR 0.20–0.28). The predictors of the second model were presence of a minor lesion, longer duration of having a previous foot ulcer and location of the previous foot ulcer. The area under the receiver operating curve was 0.76 (IQR 0.66–0.87) and the Brier score was 0.17 (IQR 0.15–0.18).

**Conclusions:**

These validated prediction models help identify those patients that are at increased risk of plantar foot ulcer recurrence and for that reason should be monitored more carefully and treated more intensively.

**Significance of this study**

**What is already known about this subject?**

- Several etiological risk factors models exist to identify variables that are associated with ulcer recurrence in high-risk people with diabetes; most risk factors are non-modifiable.
- However, most studies use inconsistent terminology to describe these models and to interpret them and are not validated.
- A validated prediction model allows more accurate assessment of ulcer recurrence risk and provides valuable information for patient follow-up and treatment.

**What are the new findings?**

- The presence of a minor lesion, living alone, increased barefoot peak plantar pressure, longer duration of having a previous foot ulcer and less variation in daily stride count are predictors of plantar foot ulcer recurrence in high-risk people with diabetes.
- Predictors of plantar foot ulcer recurrence attributed to unrecognized repetitive stress as primary biomechanical mechanism are presence of a minor lesion, longer duration of having a previous foot ulcer and location of the previous foot ulcer.
- Most predictors are variables that can be easily obtained by healthcare professionals and some predictors are modifiable factors that can be targeted for intervention.

**How might these results change the focus of research or clinical practice?**

- These prediction models allow the clinician and practitioner to timely identify patients who are at risk of developing a recurrent plantar foot ulcer and to communicate this risk with the patient.
- Additionally, they can be used to select suitable patients for therapy and guide clinician and patient in joint decision-making for preventative treatment.

## Introduction

Foot ulceration is a common and feared complication in people with diabetes mellitus; its presence has a great impact on the individuals’ quality of life, healthcare and society.^1 2^ The annual incidence of a foot ulcer in people with diabetes is approximately 2%.^3^ The risk of developing an ulcer increases if peripheral neuropathy, a history of ulceration, a foot deformity and/or peripheral vascular disease is present.^4–6^ Approximately 40% of patients who heal from an ulcer have a recurrence in the first 12 months and 60% within 3 years.^7^ This high recurrence rate is due to the many contributing factors that are still present after healing of the first ulcer, such as, neuropathy, foot deformity, increased plantar stress and peripheral vascular disease. Because of the high incidence of recurrence and subsequent risk of infection, hospital admission and amputation, a strong focus in diabetic foot disease is currently on the ‘patient in remission’ and prevention of foot ulcer recurrence.^7^ To develop adequate strategies for prevention, it is important to identify predictors of foot ulcer recurrence in diabetes.

The risk factors for diabetic foot ulcer recurrence have recently been reviewed by Armstrong **et al**.^7^ The strongest independent risk factors reported were: a vibration perception threshold >25 V,^8^ the presence of minor lesions (eg, abundant callus, blister formation or hemorrhage),^9^ the plantar location of the previous ulcer^10 11^ and the presence of peripheral vascular disease.^10^ Many risk factor models have been developed with various clinical outcomes in mind, such as ulcer recurrence.^2 8–12^ However these studies are inconsistent in description and interpretation of these models, use different starting points for patient follow-up, identify only individual etiological risk factors and are often not validated. A validated prediction model uses multiple variables to more accurately predict the risk of a future outcome, regardless of causality between the predictor and outcome.^13^

Well-designed prediction models can be of additional value in the prevention of ulcer recurrence. A prediction model allows the clinician or practitioner to timely identify patients that are at risk of developing a recurrent foot ulcer and to communicate this risk with the patient. Additionally, it can be used to select suitable patients for therapy and guides the clinician and patients in joint decision-making for preventative treatment. This applies, for example, to the frequency at which high-risk patients are screened to help identity risk and to prevent foot ulceration (once every 1–3 months is currently the recommendation for high-risk patients in international guidelines).^14^ Therefore, the aim of this study was to develop a prediction model for plantar foot ulcer recurrence in high-risk people with diabetes and to validate its predictive performance.

## Methods

### Population

Data were retrieved from a multicenter randomized controlled trial on effectiveness of custom-made footwear to prevent plantar foot ulcer recurrence.^15^ Patients were recruited between 2007 and 2010 from the multidisciplinary outpatient diabetic foot clinics of two academic and eight large general public hospitals across the Netherlands. From a total 267 possibly eligible participants, 171 people with diabetes with loss of protective sensation, a recent history of plantar foot ulceration (<18 months prior to inclusion) and newly prescribed custom-made footwear were included in this study. Loss of protective sensation was assessed using 10 g Semmes-Weinstein monofilament and biothesiometer (Biomedical Instruments, Newbury, Ohio, USA) testing.^16^ Patients were excluded if they had an active plantar ulcer, bilateral amputation proximal to the tarso-metatarsal (Lisfranc) joint, an estimated survival of <18 months and the inability to walk unaided. Participants were randomly assigned to pressure-improved custom-made footwear (~20% peak pressure relief by modifying the footwear) or non-improved custom-made footwear. Follow-up time was 18 months or until plantar foot ulceration. Written informed consent was obtained prior to inclusion from all patients.

### Potential predictors

As potential predictors of plantar foot ulcer recurrence,^9 15^ demographic, disease-related, biomechanical and behavioral factors were included.

The demographic and disease-related factors were collected at baseline through anamnesis or physical examination and included: age, gender, body mass index, diabetes type and duration, glycated hemoglobin (HbA1c), smoking (history), alcohol consumption, living alone, employment status, highest education level, vibration perception threshold, presence of peripheral arterial disease (grade I or II^17^), duration of previous ulcer(s), time between healing of the previous ulcer and study entry, location of the previous ulcer (ie, hallux, second to fifth toe, metatarsal heads or midfoot), history of amputation, severity of foot deformity and the presence of minor lesions. Foot deformity was defined as absent, mild, moderate, severe and major amputations.^9 15^ Minor lesions were defined as non-ulcerative lesions of the skin on the plantar foot, including abundant callus, hemorrhage or a blister.

The biomechanical variables assessed at study entry were barefoot plantar foot pressure (measured using an Emed-X pressure platform, Novel, Munich, Germany) and in-shoe plantar foot pressures (measured using a Pedar-X system, Novel) during comfortable level walking. Regional peak barefoot and in-shoe plantar pressure were calculated as well as two parameters that represented the cumulative load on the foot: weighted pressure (WP) and cumulative plantar tissue stress (CPTS), as described elsewhere.^9 15^

The behavioral factors assessed during the study were footwear adherence and walking activity. Adherence to wearing prescribed footwear was measured over a 7-day period using the @monitor (Academic Medical Center, Amsterdam, the Netherlands^18^). Next to overall adherence, adherence was assessed for when patients were at home and when away from home, using self-report forms. Walking activity was measured as stride count over the same 7-day period, using a StepWatch activity monitor (Orthocare Innovations, Oklahoma City, Oklahoma, USA).^9 15^ The outcome parameters were average daily stride count and day-to-day variation in stride count (ie, SD in daily stride count over a 7-day period).

For the parameters footwear adherence at home and away from home, >25% of the data were missing across subjects (namely 39.2%), and these parameters were therefore excluded as potential predictor. We used multivariate imputations for parameters with up to 25% of missing data by applying the chained equations (mice) approach as implemented by the mice package in R.^19^ This provided multiple imputations for multivariate missing data regardless of variable type, where each incomplete variable is imputed by a separate model (this is the fully conditional specification method). We used five imputation sets with a maximum of two iterations and the quick selection of predictor option, which is useful when there are many variables. Little’s missing completely at random test^20^ failed to show potential patterns in missing data (χ^2^=58.57, df=49, p=0.16).

### Primary outcome

The primary outcome was plantar foot ulcer recurrence in 18 months. Foot ulcer was defined as a full-thickness lesion of the skin, irrespective of duration.^14 17^ Recurrence was defined as an ulcer at the same location as the previous one, or at any other plantar location on the ipsilateral or contralateral foot. If a patient, treating physician, or other healthcare provider (eg, podiatrist) identified an ulcer during follow-up, they were instructed to report the lesion, complete a foot ulcer form and have photographs of the lesion taken. During 3-monthly follow-up visits, patients were asked about any lesion that had occurred and electronic patients files were checked for any unreported ulcer. Outcome assessment was done blinded by three independent diabetic foot experts who assessed photographs of the plantar foot if an ulcer was suspected. Two additional foot experts were consulted when unanimity was not reached.

### Model development

Two logistic regression prediction models of plantar foot ulcer recurrence were developed. The first model was on prediction of all recurrent plantar foot ulcers in the study. The second model was on prediction of those recurrent plantar foot ulcers that were suggested to be the result of unrecognized repetitive stress. This was defined as an ulcer occurring at the same location as the previous ulcer and not being the result of a traumatic event, as reported by the patient. This division in models was analogous to the study by Waaijman **et al**.^9^ Dependent on the prediction model, the foot with the worst outcome for a given parameter with bilateral outcomes was chosen (first model), or the foot where the previous ulcer was located (second model). Reporting on the development of these models was done according to the Transparent Reporting of a multivariable prediction model for Individual Prognosis Or Diagnosis statement.^21^

Based on clinical reasoning, knowledge from the literature and clinical feasibility in assessment, we considered all the above-mentioned potential predictors as variables in the model. Potential predictors that showed to be strongly correlated with each other (ie, correlation coefficient >0.5) contribute little independent information to the model. Using clinical reasoning regarding which potential factor to exclude, we excluded the following variables based on high intervariable correlation: age, HbA1c and type of diabetes (all correlated with duration of diabetes), education (correlated with living alone) and average daily stride count (correlated with day-to-day variation in stride count). Both WP and CPTS were excluded from the model, because they strongly correlated with each underlying factor in these composite variables.

### Model fitting and validation

The model development strategy went through four stages: (a) creating five imputed datasets with no missing values, (b) further variable selection in each imputed dataset, (c) fitting a logistic regression model on each of the five imputed datasets to predict ulcer outcome based on these variables and (d) pooling these five models into a final prediction model. The final logistic regression model for plantar foot ulcer recurrence will be represented by its linear predictor (LP). The predicted probability can be calculated from this LP with the following formula: 1/(1+*e*^−LP^).

Further variable selection (stage ‘b’ above) was deemed important because after initial expert selection of variables many potential predictors remained and the dataset of 171 patients is relatively small. Definitive variables for the model were selected in two steps. First, we selected variables that had a univariable association with the primary outcome with a p value <0.2. Second, we developed a multivariable model with those selected variables and used backward variable selection based on the Akaike Information Criterion (AIC)^22 23^ aiming at finding the optimal set of predictors. By giving a penalty for model complexity (in terms of the number of included variables), the AIC strikes a good balance between the likelihood of the model (which always increases with the number of included variables) and its complexity (the more complex the model, the more likely it would overfit the data).

We used 10-fold cross-validation to internally validate the prediction strategy. This means that the whole model development strategy (including the five multiple imputation datasets and the variable selection process) is repeated in each of the 10 folds on the training set (90% of the data) and tested on the 10% held-out dataset of that fold.

### Model performance

Model performance was assessed in terms of discrimination and calibration.^24^ Discrimination was measured for all five pooled models and the final prediction model by the area under the receiver operating curve (AUC) using the median, IQR and minimum and maximum over 10 folds.^25^ The AUC curves of the final models are also presented. It refers to the ability of the model to provide a higher probability of the event (ie, ulcer recurrence) to those patients with the event than those without the event. The higher the value of the AUC the better the discrimination ability. Calibration refers to the closeness of the predicted probabilities to the true ones as estimated by appropriate patient groups, and was assessed using calibration graphs. The Brier score,^26^ which is the mean squared error of a prediction, combines both elements of discrimination and calibration and was also assessed for all five models on the imputed datasets and the final pooled prediction model (median, IQR and minimum and maximum over 10 folds). A Brier score ranges from 0 to 1 and if the predicted values by the model and the observed values are completely concordant then the Brier score is 0. Finally, the positive predictive value (PPV), the proportion of positive results that are truly positive, was calculated in each fold when the threshold was set at the 75th percentile of predictions.

We used the average predictive comparison to assess the change on the probability of the outcome due to the change in each predictor in the model, hence indicating the influence of each of the individual predictors on the probability of ulcer recurrence when all other predictors remain constant.^27^ Descriptive statistics were performed using SPSS V.22.0 software (IBM, Armonk, New York, USA). All model analyses were performed in the R statistical environment (R Foundation for Statistical Computing for Windows V.2.9.0 (http://www.R-project.org)).^28^

## Results

Table 1 describes the characteristics of the study sample. Of the total 171 patients, 141 were male and the mean age was 63.3 years. Seventy-one patients (=42%) had a recurrent ulcer with a mean time to ulceration of 197 days. Forty-one of those 71 patients (=24% of the total group) had a recurrent ulcer due to unrecognized repetitive stress, with a mean time to ulceration of 173 days.

**Table 1:** Characteristics of the study

Potential predictor	Outcome	Missing values N (%)
Age (years)	63.3±10.1	
Male	141 (82.5)	
Body mass index (kg/m^2^)	30.7±5.7	
Smoking or history of smoking	114 (66.7)	2 (1.2)
>2 units alcohol intake per day	20 (11.7)	1 (0.6)
Living alone	46 (26.9)	
Education		
Low	98 (56.1)	
Medium	31 (18.1)	
High	44 (25.7)	
Employed	37 (21.6)	
Type of diabetes		
1	49 (28.7)	
2	122 (71.3)	
Years of diabetes	17.3±13.5	2 (1.2)
Glycated hemoglobin (%)	7.58±1.44	9 (5.3)
Months duration of previous ulcer	8.7±13.3	7 (4.1)
Daily stride count	3359±1749	15 (8.8)
Variation in daily stride count	1194±713	15 (8.8)
Adherence (%)	72.8±24.3	20 (11.7)
Adherence (%) at home	62.4±32.4	67 (60.8)
Adherence (%) away from home	87.8±26.5	67 (60.8)
Previous ulcer location		
Hallux	41 (24.0)	
2nd to 5th toe	34 (19.9)	
Metatarsal heads	91 (53.2)	
Midfoot	5 (2.9)	
History of amputation	65 (38)	
Foot deformity		
Absent	6 (3.5)	
Mild	55 (32.2)	
Moderate	77 (45.0)	
Severe	27 (15.8)	
Major amputation	6 (3.5)	
Minor lesions at entry	60 (35.1)	
Peripheral arterial disease		4 (2.3)
Grade 1	93 (54.4)
Grade 2	74 (43.3)	
Vibration perception threshold (Volt)	47.5±8.2	
Months between healing of previous ulcer and study entry	5.0±5.5	8 (4.7)
Improved custom-made footwear	85 (49.7)	
Barefoot peak plantar pressure forefoot (kPa)	1029±257	4 (2.3)
Barefoot peak pressure at previous ulcer location (kPa)	726±396	24 (14.0)
In-shoe peak pressure forefoot (kPa)	275±78	1 (0.6)
In-shoe peak pressure at previous ulcer location (kPa)	186±94	21 (12.3)
In-shoe peak pressure forefoot <200 kPa and adherence >80%	6 (3.5)	22 (12.9)

*Data are expressed as number (%) or mean±SD.

### Model 1: all recurrent plantar foot ulcers

The model for this outcome contained five predictors (table 2): increased barefoot peak plantar pressure at the forefoot (in kPa), presence of a minor lesion, duration of the previous ulcer in months and living alone were positive predictors for recurrence; a higher variation in day-to-day stride count (in SDs) was a negative predictor of recurrence.

**Table 2:** Predictors for model 1 (all recurrent plantar foot ulcer) and model 2 (plantar foot ulcer recurrence from unrecognized repetitive stress)

Predictor	Coefficient	95% CI	Change in variable	Change in ulcer probability
*Model 1: all plantar foot ulcer recurrences*				
Intercept	−2.1	−3.8 to −0.37		
Living alone	0.76	0.015 to 1.5	No to yes	0.16
Minor lesions	1.4	0.69 to 2.1	No to yes	0.25
Duration of the previous ulcer	0.034	0.0026 to 0.065	12 months	0.085
Barefoot peak plantar pressure	0.0013	−0.00013 to 0.0027	255 kPa	0.07
Variation in daily stride count	−0.047	−0.10 to 0.0098	700 steps	−0.065
*Model 2: plantar foot ulcer recurrence from unrecognized repetitive stress*				
Intercept	−1.8	−2.5 to −1.1		
Minor lesions	2.2	1.3 to 3.1	No to yes	0.37
Duration of the previous ulcer	0.038	0.0047 to 0.071	12 months	0.064
Previous ulcer location:				
Metatarsal heads	Reference			
Hallux	−1.6	−2.8 to −0.4	In comparison to patients with an ulcer at the metatarsal heads	−0.028
Toes	−2.0	−3.6 to −0.4		−0.21
Midfoot	0.024	−2.3 to 2.4		0.21

CI, Confidence interval.

The LP of the logistic regression model for recurrent plantar foot ulcer was: −2.1+0.76×living alone+1.4×minor lesion present+0.034×duration of previous ulcer in months+0.0013×barefoot peak plantar pressure at the forefoot in kPa–0.047×variation in daily stride count in SDs.

Table 2 also shows the average predictive comparison for model 1. If a patient has a minor lesion present or lives alone there is a 0.25 and 0.16 higher probability, respectively, for ulcer recurrence. If a patient has a duration of past ulceration of 12 months or an increase in barefoot peak plantar pressure of 255 kPa, there is a higher probability of 0.085 or 0.07, respectively, for ulcer recurrence. An increase of variation in day-to-day stride count of 700 steps decreases the probability for ulcer recurrence with −0.065.

**Figure 1:**
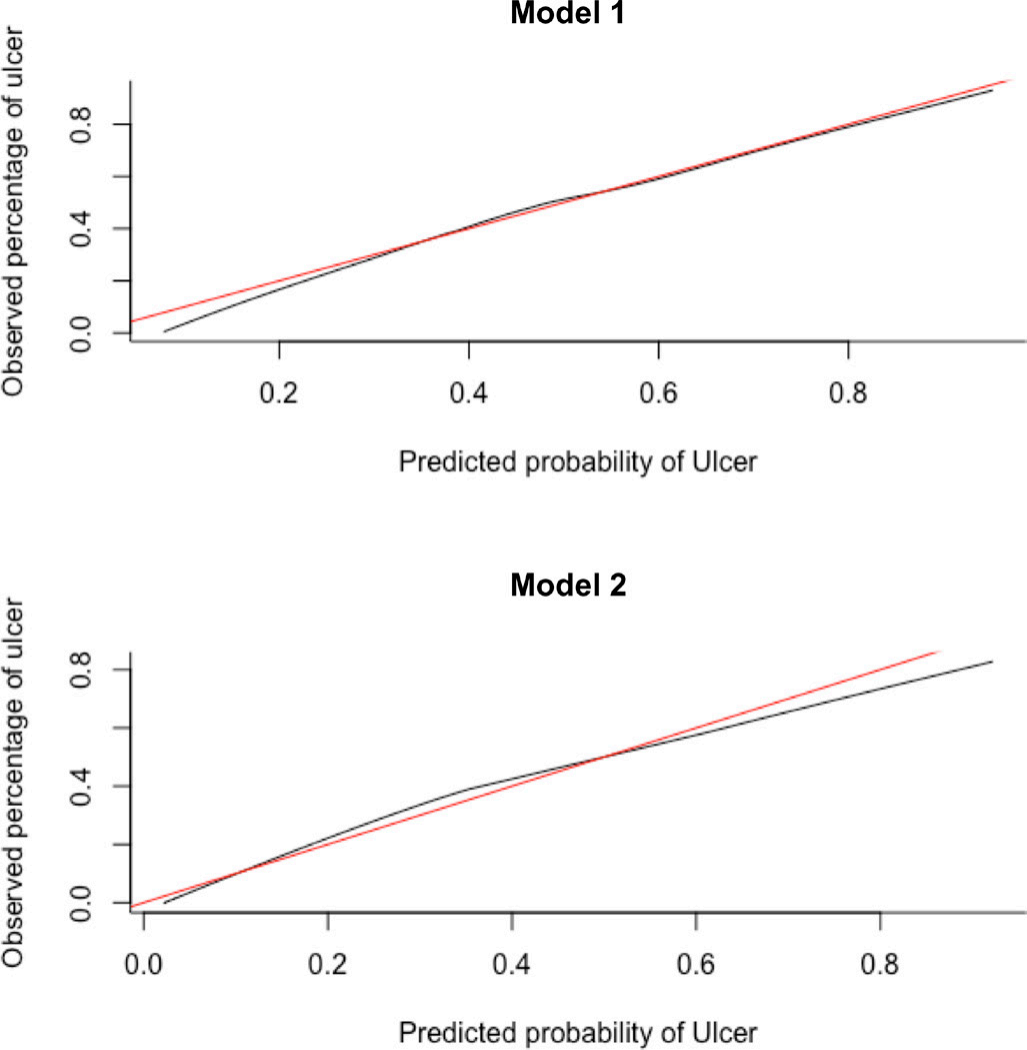
Calibration graphs for model 1 (all recurrent plantar foot ulcer) and model 2 (plantar foot ulcer recurrence from unrecognized repetitive stress). In each graph the black line shows the observed proportion of the event versus the probability of the event as predicted by the model. Ideally all the points fall on the diagonal red line.

**Figure 2:**
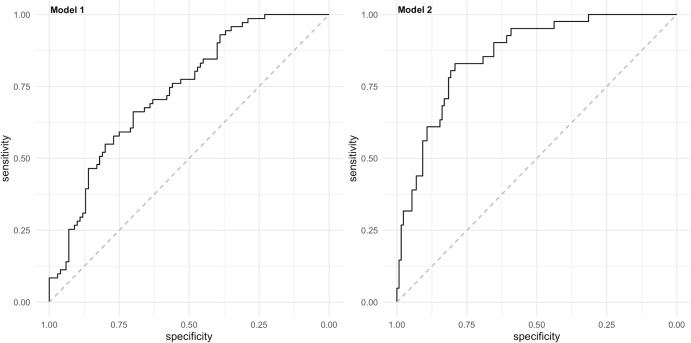
Area under the receiver operating curve for model 1 (all recurrent plantar foot ulcer) and model 2 (plantar foot ulcer recurrence from unrecognized repetitive stress).

Figure 1 shows the calibration graph based on the average predictions per patient of the final model on the five imputation datasets. The graph shows that the predicted probability of a recurrent ulcer and the observed number of recurrent ulcers agreed over almost the whole range of probabilities. Only when the predicted probability is lower than 0.35, the prediction slightly underestimates the proportion of observed recurrent ulcers. Figure 2 shows the AUC of the final model. The median AUC of this final model was 0.68 (IQR 0.61–0.80). The minimum AUC was 0.53 and the maximum AUC was 0.89 over the 10 folds with a SD of 0.159. The median Brier score was 0.24 (IQR 0.20–0.28). The median PPV was 65% (IQR 50%–79%).

### Model 2: plantar foot ulcer recurrence from unrecognized repetitive stress

The model for this outcome contained three predictors (table 2): presence of a minor lesion, duration of the previous ulcer in months and the location of the previous ulcer.

Based on these results, the LP of the logistic regression model was: −1.8+2.2×minor lesion present+0.038×duration of previous ulcer in months+−1.6×ulcer location ‘hallux’; −2.0×ulcer location ‘lesser toes’; 0.024×ulcer location ‘midfoot’. For this formula, ulcer location under the metatarsal heads was the reference category.

The average predictive comparison is shown in table 2. If a patient has a minor lesion or a duration of past ulceration of 12 months, there is a 0.37 and 0.064 higher probability, respectively, for ulcer recurrence. If the previous ulcer was located on the plantar hallux or toes, the probability for ulcer recurrence decreased with −0.028 and −0.21, respectively compared with patients who had the previous ulcer under the metatarsal heads. However, if the previous ulcer was located under the midfoot, the probability increased with 0.21 compared with patients with a previous ulcer under the metatarsal heads.

Figure 1 shows the calibration graph based on the average predictions per patient for the model on the five imputation datasets. The graph shows that the predicted probability of ulcer recurrence from unrecognized repetitive stress slightly overestimates the observed proportion of recurrent ulcers from unrecognized repetitive stress when the predicted probability is between 0.10 and 0.50 and slightly underestimates the observed proportion of recurrent ulcers from unrecognized repetitive stress when the predicted probability is higher than 0.50. Figure 2 shows the AUC of the final model. The median AUC of this final model was 0.76 (IQR 0.66–0.87). The minimum AUC was 0.50 and the maximum AUC was 0.88 over 10 folds with a SD of 0.175. The median Brier score was 0.17 (IQR 0.15–0.18). The median PPV was 65% (IQR 50%–79%).

## Discussion

This study showed that presence of a minor lesion, living alone, increased barefoot peak plantar pressure, longer duration of having a previous foot ulcer and less variation in daily stride count are predictors of plantar foot ulcer recurrence in high-risk people with diabetes. The prediction model showed relatively poor discrimination but had good calibration. Presence of a minor lesion and longer duration of having a previous foot ulcer were also predictors of plantar foot ulcer recurrence attributed to unrecognized repetitive stress, in addition to location of the previous foot ulcer. The model showed fair discrimination and reasonable calibration.

The first prediction model contains a combination of biomechanical, behavioral, patient-related and disease-related factors; the second model only includes biomechanical and disease-related factors. The fact that both models include biomechanically related factors is because we focus on foot ulcers on the plantar surface, which have a stronger biomechanical etiology than non-plantar foot ulcers.^7^ The presence of a minor lesion was in both models a predictor, showing the largest observed change in ulcer recurrence probability of all predictors. This is in accordance with the study by Waaijman *et al*, who showed on the same data set that presence of a minor lesion was the strongest associated factor with plantar ulcer recurrence.^9^ Minor lesions such as abundant callus and blisters are the result of mechanical stress and are therefore amendable through pressure-relieving footwear. Furthermore, they allow early identification of impending ulceration that helps to inform the patient about risk and helps to reduce ulcer recurrence risk if treated appropriately.^15 29^

Living alone predicts plantar foot ulcer recurrence in our first model. This suggest that partners or relatives are important in helping to preserve the patient’s foot health. Social status and its association with ulcer recurrence was previously investigated, but has not before shown to be a significant one.^9 30^ Variation in stride count negatively predicted ulcer recurrence in the first model, suggesting that less variation in daily stride count predicts recurrence. This is contrary to the study by Armstrong **et al**, who found in medium-risk to high-risk patients that a higher variability in daily stride count increases risk of ulceration.^31^ They postulate that high-risk patients are less able to withstand repetitive stress and that modulating the ‘peaks and valleys’ of their daily stride activity might reduce ulcer recurrence risk.^31^ These authors also showed that daily stride count in patients who ulcerated was significantly lower than in those who did not, an outcome that was not found in our data.^9^ This sounds counterintuitive given the lower cumulative stress exerted on the foot in these non-ulcerated cases, but suggestions that biomechanical loading of the foot leads to tissue adaptation and improved load tolerance,^32 33^ supports these findings. More research is needed to untangle the apparent complex interaction between amount of daily activity and risk of plantar foot ulcer recurrence.

The location of the previous foot ulcer predicted recurrence in our second model. The probability of developing an ulcer at the same location was lower for a previous ulcer at the hallux compared with one at the metatarsal heads, and even lower for a previous ulcer at the lesser toes. The probability of ulcer recurrence at the midfoot was high, likely because all patients with a midfoot ulcer had Charcot midfoot deformity. In general, a plantar location of a previous ulcer increases risk of ulcer recurrence.^9 11^ Peters *et al* found that plantar hallux ulcers are more prone to recurrence than any other ulcer (plantar or dorsum).^10^ The distribution of plantar pressures over the foot likely explains our results, where highest pressures are generally found at the metatarsal heads, followed by the hallux and then the lesser toes.^34^ Offloading these high-risk areas can help in reducing ulcer recurrence risk.^15 35^

Most predictors identified in both models are variables that can be easily and readily obtained by healthcare professionals through anamnesis, physical examination and measurement. Only barefoot plantar pressure analysis is not easily obtained in every setting, although its use is increasing, and the need for such measurements is indicated in this and other studies. For the purpose of clinical practice, it is possible to integrate these models in an electronic healthcare system that can provide predictive risk when data input based on anamnesis and physical examination is completed. When using both models, the treating physician should be aware that the first model slightly underestimates the risk in patients at a low risk of ulcer recurrence, while the second model slightly overestimates the patients at low risk of ulcer recurrence and slightly underestimates the patients at high risk of ulcer recurrence. Based on the second model, it might therefore be possible that patients with a high predicted probability of ulcer recurrence may be treated or seen less frequently than they supposed to be based on the actual probability of ulcer recurrence.

However, while accurate predictions give valuable insight into which patients are at a high risk of developing plantar foot ulcer recurrence and need more frequent follow-up, the coefficients in our prediction models are mainly useful for implementing these models by others (eg, for external validation). They should not be interpreted causally, and due to possible correlations between them odds ratios might not be meaningful. Nevertheless, some predictors are modifiable factors that can be targeted for intervention using current literature and clinical knowledge. Minor lesions, for example, can be treated on sight and peak plantar pressures can be reduced by limiting barefoot walking.^35^ Advice regarding an appropriate and safe level of daily activity is also possible.^35^ It is important, however, to stress that it is unclear what effect these interventions will have on the predicted risk of plantar foot ulcer recurrence.

Several strengths and limitations apply to this study. We used the same dataset as Waaijman **et al**; however, their models are etiological in nature and aim to explain whether an ulcer recurrence can reliably be attributed to a risk factor. Missing data were not accounted for by these authors, which may lead to bias. In our models, missing data were multiply imputed. Additionally, their study lacked internal validation; the reported sensitivity of 81% and specificity of 50% are likely overestimated. Another strength of our study is that we used AIC and cross-validation for the selection of potential predictors while other studies used a multivariate regression analysis with significant factors (p<0.10) from a univariate analysis.^2 8–11^ Also, most studies do not or only partly report the performance of their models in terms of discrimination and calibration.^2 8–11^

A first and important limitation is the limited number of patients included in our database. With only 71 and 41 events for model 1 and 2, respectively, only a small number of predictors is warranted in the model in order to avoid overfitting. Because we have many candidate predictors, the choice of predictor set is not very stable and other predictors could be selected when having other samples of the same size. However, we relied on clinical knowledge for the initial selection of variables, then we used a liberal p value of 0.2 for the second stage and then used the AIC to select the remaining variables. Second, the outcome of the second model was partly based on the patient’s self-report that an ulcer was not a result of an acute trauma, which might introduce a recall bias. Third, some variables had too much missing data that prevented us from including them in the model. Finally, external validation of our model on another database to evaluate model performance in other high-risk patients with diabetes was not performed.

## Conclusion

We provided well-designed and internally validated prediction models for risk of plantar foot ulcer recurrence in high-risk people with diabetes. The model predicted recurrence based on presence of a minor lesion, living alone, increased barefoot peak plantar pressure, longer duration of having a previous foot ulcer and less variation in daily stride count, with good calibration but relatively poor discrimination. The model for repetitive stress ulcers predicted recurrence based on presence of a minor lesion, longer duration of having a previous foot ulcer and the location of the previous ulcer, with fair discrimination and a reasonable calibration. These models help identify those patients that are at increased risk of plantar foot ulcer recurrence and for that reason should be monitored more carefully and frequently and treated more intensively.
